# Glycemic Profiles of Healthy Individuals with Low Fasting Plasma Glucose and HbA1c

**DOI:** 10.5402/2011/435047

**Published:** 2011-12-08

**Authors:** Kyoko Nomura, Tomoyuki Saitoh, Gwang U. Kim, Toshikazu Yamanouchi

**Affiliations:** ^1^Department of Hygiene and Public Health, School of Medicine, Teikyo University, Kaga 2-11-1, Itabashi, Tokyo 173-8605, Japan; ^2^Faculty of Pharmaceutical Sciences, Teikyo University, Kaga 2-11-1, Itabashi, Tokyo 173-8605, Japan; ^3^Division of Internal Medicine, Nishi-Arai Hospital, Nishiarai honcho 5-7-14, Adachi, Tokyo 123-0845, Japan; ^4^Department of Internal Medicine, Teikyo University Hospital, Kaga 2-11-1, Itabashi, Tokyo 173-8605, Japan

## Abstract

Scant data exists on glucose profile variability in healthy individuals. Twenty-nine healthy subjects without diabetes (86% male; mean age, 38 years) were measured by a CGM system and under real-life conditions. The median percentage of time spent on the blood glucose >7.8 mmol/L for 24 hrs was greater than 10% in both NFG and IFG groups. When subjects were divided into either NFG group (i.e., FPG levels of <5.6 mmol/L; *n* = 22) or IFG group (FPG levels of 5.6–6.9 mmol/L; *n* = 7), all CGM indicators investigated but GRADE scores, including glucose variability measures, monitoring excursions, hyperglycemia, hypoglycemia, and 24-hour AUC, did not differ significantly between the two groups. GRADE score and its euglycemia% were significantly different between the two groups. Among various CGM indicators, GRADE score may be a sensitive indicator to discriminate glucose profiles between subjects with NFG and those with IFG.

## 1. Introduction

Literatures suggest that the extent of glucose levels may be related to the subsequent development of diabetes even among healthy individuals. For example, two large-scale epidemiological studies have demonstrated a clear diabetes risk gradient in glycemic levels within what was previously considered the “normal” profile (i.e., an fasting plasma glucose (FPG) level of 100 mg/dL and a Hemoglobin A1c (HbA1c) level of 6.0% [1, 2]). In addition to the evidence that the strong independent association is observed between the level of normal fasting plasma glucose (NFG) and the incidence of diabetes, another study demonstrated the powerful effects of impaired fasting glucose (IFG) or impaired glucose tolerance (IGT) on the development of diabetes [3]. These literatures suggest that glucose profiles in healthy individuals may be different depending on glycemic categories such as normal fasting glucose, IFG, and IGT. 

In this study, we investigated differences in glycemic profiles between healthy subjects with NFG and those with IFG by utilizing a continuous glucose monitoring (CGM) system. The CGM system (Medtronic Minimed, Northridge, Calif), a valid and reliable, minimally invasive system, enables the analysis of various glucose patterns that are not assessable by conventional glucose indicators [4]. A recent CGM study demonstrated that in a group of healthy individuals without diabetes selected on the basis of a very low baseline FPG level, 10% of subjects registered glucose levels that were considered to be “prediabetic” or indicative of a considerable period of IGT [5]. Given than few data exist on glucose variability in healthy individuals, the result of the present study may help us understand what constitutes normal glucose profile.

## 2. Materials and Methods

### 2.1. Subjects and Materials

Thirty-seven healthy adult Japanese subjects, who were medical doctors and pharmaceutical sales representatives, were recruited to this study. The study subjects were further selected on the basis of (1) having no history of diabetes, hypertension, and metabolic syndrome and (2) a plasma glucose level of <7 mmol/l after an overnight fast, HbA1c level of <6.0%, or plasma 1,5-anhydroglucitol (1,5-AG) level of >14.0 *μ*g/mL. Accordingly, 29 subjects were enrolled in this study after they provided written informed consent. The study protocol was approved by the institutional review board of Nishi-Arai Hospital.

### 2.2. Clinical Evaluation and Laboratory Measurements

Plasma glucose level was measured using the hexokinase-G6PD method (Denka Seiken, Niigata, Japan). Plasma 1,5-AG level was determined with an autoanalyser system (Automatic Clinical Analyzer, Model 7150 Hitachi Tokyo, Japan) using a modified column enzymatic test. Serum insulin values were measured using a chemiluminescent enzyme immunoassay. Homeostasis of minimal assessment of insulin resistance (HOMA-IR) was calculated from the FPG level and the immunoreactive insulin (IRI) level using the formula: HOMA-IR = [FPG  (mmol/l) × IRI  (*μ*U/mL)]/22.5.

A borderline level of insulin resistance was defined as a HOMA-IR score of ≥1.6, and definite insulin resistance as a HOMA-IR score of ≥2.5. Pancreatic beta-cell function (i.e., HOMA-B) was quantified using the equation: HOMA-B = IRI  (*μ*U/mL) × 20/[FPG  (mmol/mL) − 3.5].

HbA1c level was measured by immunoassay using monoclonal antibodies (DM-JACK; Kyowa Medex, Tokyo, Japan). Because the measurement range for HbA1c was in accordance with the guidelines of the Japan Diabetes Society, results were converted into National Glycohemoglobin Standardization Program (NGSP) values by adding 0.4% based on the equation: NGSP value (%) = JDS value (%) + 0.4%.

### 2.3. CGM System Measurement and Indicators

MiniMed CGM system sensors were inserted into subjects for 48 hrs. Finger stick glucose levels were measured every 8 hrs for calibration. During CGM, no attempt was made to standardize participant behavior, for example, by controlling dietary calorie intake or exercise. Instead, subjects were asked to modify their usual daily behavior as little as possible.

The CGM measures investigated were mean blood glucose (MBG), standard deviation (SD), largest amplitude of glycemic excursion (LAGE), mean amplitude of glycemic excursion (MAGE) [6], the *M*-value [7], mean of daily difference (MODD) [8], the *J*-index [9], high blood glucose index (HBGI) [10], low blood glucose index (LBGI) [11], average daily risk range (ADRR) [12], Glycemic Risk Assessment Diabetes Equation score: GRADE score (hypoglycemia%, euglycemia%, hyperglycemia%) [13], percentage coefficient of variation (%CV) [14], hypoglycemic index [14], hyperglycemia index [14], index of glycemic control (IGC) [14], continuous overall glycemic action (CONGA), and 24-hr-area under the curve (AUC). Each measure is briefly defined in Table 1 and has been described elsewhere [14]. The *M*-value, a glucose swing measure, was computed when the average blood glucose was 5.6 mmol/L [7]. The GRADE score was based on clinicians' value judgments of relative importance of hypo- and hyperglycemia and summarizes diverse glycemic profiles to a single assessment of risk. In reporting a GRADE score, percentages are used to describe weighted risks calculated from hypoglycemia%, euglycemia%, and hyperglycemia% [13]. For example, a GRADE score of 10 (18%, 80%, 2%) indicates an increased glycemic risk with a risk contribution coming from hypoglycemic episodes. Conversely, a GRADE score of 10 (2%, 48%, 50%) would be typical of someone with poorly controlled hyperglycemia. According to a previous report [13], a GRADE score of <5 reflects the euglycemic range.

The AUC for 24 hrs was measured by using the trapezoidal method [15]. The percentages of time (PT) spent on the blood glucose <3.3 mmol/L, >7.8 mmol/L, >8.9 mmol/L, and >10 mmol/L for 24 hrs were computed as AUCs attributable to these ranges divided by AUC for 24 hrs (Table 1).

### 2.4. Statistical Analyses

The distribution of each glycemic indicator was evaluated for normality using the Shapiro-Wilk test. If values were normally distributed, the mean value was presented; if not, the median was presented. Study subjects were divided into two groups: those with FPG levels of <5.6 mmol/L (i.e., NFG group; *n* = 22) and those with FPG levels of 5.6–6.9 mmol/L (i.e., IFG group; *n* = 7). Differences were evaluated using the chi-square test or Fisher's exact test for categorical variables and the *t*-test or the Wilcoxon rank sum test for continuous variables according to Shapiro-Wilk test results. Depending on the distribution of each variable, Pearson or Spearman correlation coefficient was calculated for the association between the significant CGM indictors and conventional glycemic indicator (i.e., HbA1c and 1,5-AG). All analyses were conducted using SAS software Version 9.12 (Cary, NC, USA). A value of *P* of <0.05 was considered to be statistically significant.

## 3. Results

### 3.1. Subject Baseline Characteristics

Twenty-nine subjects were 86% male, and had a mean ± SD of age 38 ± 6.9 years, body mass index 25 ± 2.3 kg/m^2^, HbA1c level 5.2 ± 0.2%, and 1,5-AG level 25.7 ± 6.5 *μ*g/mL. When subjects were divided into two FPG groups, the proportions of male subjects were 82% versus 100% (*P* = NS, Fisher's exact), median insulin levels were 5.8 *μ*U/mL versus 11.5 *μ*U/mL (*P* = 0.021), HOMA-IR was 1.3 versus 3.5 (*P* = 0.007), HOMA-beta was 68 versus 60 (*P* = NS), HbA1c levels were 5.2% versus 5.4%: *P* = NS, and 1,5-AG levels were 27 *μ*g/mL versus 22 *μ*g/mL (*P* = 0.001). Thus, subjects with IFG appeared to have higher levels of insulin and insulin resistance compared with those with NFG, but retained pancreatic beta-cell function.

### 3.2. CGM Indicators of Healthy Subjects without Diabetes

The CGM indicators according to FPG levels are shown in Table 2. Among CGM measures, including glucose variability measures, monitoring excursions, hyperglycemia, hypoglycemia, and 24-hour AUC, GRADE score and euglycemia% were statistically different (*P* = 0.022 and *P* = 0.044, resp.) between subjects with NFG group and those with IFG group. GRADE scores were 2.7 (0%, 70%, 30%) in NFG group versus 3.9 (10%, 40%, 50%) in IFG group. Even though both groups had euglycemia referring to a previous literature [13], GRADE score was significantly higher in IFG group than in NFG group and euglycemia% was significantly higher in NFG group than in IFG group. 

Except for GRADE score, the remaining CGM indicators did not differ significantly between the two groups. Means or medians of these CGM measures fell in the normal ranges for healthy individuals or individuals with stable diabetes [6–8, 12–14, 16, 17]. The median percentage time within a 24-hour period that the blood glucose level was >7.8 mmol/L was greater than 10% in both NFG and IFG groups. This means that approximately 10% of CGM for 24 hrs in both two groups reached the glucose tolerance threshold of 7.8 mmol/L at some time during the 24-hour monitoring period.

The median percentages of time within a 24-hour period in NFG group that the blood glucose levels were >7.8 mmol/L, >8.9 mmol/L, and >10 mmol/L were 13% (3%, 23%), 1% (0%, 6%), and 0% (0%, 1%), respectively. When calculated on a head-count basis in the same group (i.e., NFG group), AUC above 7.8 mmol/L was observed in 19/22 subjects, and AUC above 8.9 mmol/L in 12/22 subjects, and AUC above 10 mmol/L in 7/22 subjects at some point within 24 hrs.

### 3.3. Correlation Coefficients between GRADE Score and HbA1c and 1,5-AG

Table 3 shows the correlation coefficients between GRADE score (hyopglycemia%, euglycemia% hyperglycemia%), and HbA1c and 1,5-AG in the entire subject. GRADE score and euglycemia% were highly correlated with 1,5-AG (*r* = −0.583 and *P* = 0.001, and *r* = 0.512 and *P* = 0.005, resp.) and moderately correlated with HbA1c (*r* = 0.450 and *P* = 0.014, and *r* = −0.191 and *P* = 0.321, resp.).

## 4. Discussion

This study demonstrated that GRADE score and euglycemia% significantly differed between subjects with NFG and those with IFG, although GRADE score was less than 5 in both groups, suggesting glucose profiles in both groups were euglycemia. Furthermore, GRADE score and euglycemia% were highly correlated with 1,5-AG in this study. Previous literatures suggest that 1,5-AG is an indicator of glycemic variability [18, 19]. Previously Yamanouchi et al. [18] reported that 1,5-AG levels manifested greater fluctuation of plasma glucose even though the mean plasma glucose and HbA1c levels suggested good control. Furthermore, Kishimoto et al. [19] suggest that the plasma 1,5-AG concentration can be a useful index of the daily excursion of blood glucose, especially in patients with well-controlled diabetes. In our study, CGM indicators such as MAGE, M-value, and LARGE that reflect glucose excursion or fluctuation were not significantly different between the two FPG groups. However, we found that there was significant correlation between GRADE score and 1,5-AG. This may suggest that a GRADE score may be a sensitive CGM indicator to discriminate glucose profiles in subjects with NFG from those in subjects with IFG.

Except for GRADE score, the remaining CGM indicators, including glucose variability measures, monitoring excursions, hyperglycemia, hypoglycemia, and 24-hour AUC, did not differ significantly between subjects with NFG and those with IFG. In fact, means or medians of these CGM measures in both groups fell in the normal ranges for healthy individuals and individuals with stable diabetes [6–11, 13, 14, 16, 20–22] suggesting that glucose profile in healthy individuals without diabetes was less variable than those for diabetics.

Although glucose variability detected by CGM was found to be small, we found that more than 10% of the 24-hour AUC was above 7.8 mmol/L and 1% of the 24-hour AUC above 8.9 mmol/L, even in subjects with FPG levels of <5.6 mmol/L. Indeed, the plasma glucose level, at some point within a 24-hour period, exceeded the IGT threshold of 7.8 mmol/L in nearly all individuals who belonged to NFG group, exceeded 8.9 mmol/L in half of subjects, and exceeded 10 mmol/L in one-third of subjects. A 2010 ADA reports that healthy individuals with FPG levels of <5.6 mmol/L have no risk of future cardiovascular disease complications in diabetes and do not require any intervention [23]. However, it requires a justification. 

For example, a large clinical trial revealed that, even in individuals with FPG and 2-hour plasma glucose (2hPG) levels within the normoglycemic range, higher 2hPG was associated with insulin resistance and increased cardiovascular disease mortality [24]. In view of the evidence that the glycemic profile in healthy individuals is related to the pathogenesis of cardiovascular complications associated with abnormal glucose metabolism, the accuracy of the current diagnostic practice such as self-monitoring of blood glucose must be improved in order to capture abnormal glycemic profiles. In this regard, CGM in healthy individuals may not be acceptable for screening a larger population for risk of future diabetes due to the costs involved and resources needed. However, we found that 1,5-AG level was significantly correlated with GRADE score that was the only significant CGM indicator. Thus, it is suggested that 1,5-AG may be a useful alternative for CGM to discriminate glucose profiles between healthy individuals with NFG and those with IFG. Future studies with a larger sample size will be required to verify the results of the present study.

In addition to small number of study subjects, our study has several limitations. First, since a 75-gram oral glucose tolerance test (OGTT) was not performed, we might have had diabetes at inclusion. According to a previous report, because FPG alone fails to diagnose 30% of diabetes who were diagnosed by a 2-hour plasma glucose [25]. In addition, IGT can be easily missed as oriental population are often characterized by postprandial hyperglycaemia. Second, FPG levels in this study were assessed only once at baseline. The inter- and intracoefficients of variations in glucose values may have caused some random misclassification of subjects into the two FPG categories and thereby influenced our results. Third, although the measurement was performed under real-life conditions, lifestyle-related factors that influence glucose levels, such as exercise, sleep, and stress including emotional and physical stress, were not examined. Fourth, 85% of our study subjects were male, which might affect the results. Finally, this study assessed patient at risk of diabetes by using FPG and HbA1c alone. Thus, the results of our study should be interpreted with caution.

## 5. Conclusion

Despite these limitations, even under real-life conditions, the glucose profiles of healthy individuals, including subjects with NFG and IFG, are far less variable than those of diabetic individuals [6–11, 13, 14, 16, 20, 21]. Nevertheless, nearly all individuals with FPG levels of <5.6 mmol/L exceeded the impaired glucose tolerance threshold of 7.8 mmol/L at some point within a 24-hour period, indicating that glucose levels in healthy individuals frequently reach the impaired glucose tolerance threshold. Although most CGM measures did not differ significantly between the two FPG groups, the GRADE score may be a sensitive indicator that distinguishes the glucose profiles of subjects with NFG from those of subjects with IFG.

## Figures and Tables

**Table 1:** CGM indicators.

Glycemic measure	Brief definition	Reference
MBG	Mean of all glucose values	5.7 mmol/L [20], 5.8 mmol/L [26] for healthy individuals
SD	SD of all glucose values	1 mmol/L [20], 0.8 mmol/L [26] for healthy individuals
LAGE	Blood glucose (BG)_max_-BG_min_	1.4–5.7 mmol/L for healthy individuals [16]
MAGE	Average amplitude of upstrokes or downstrokes with magnitude greater than 1 SD	1.2–3.3 mmol/L for healthy individuals [6]
*M*-value	Weighted average of glucose values, with progressively larger penalties for more extreme values	0 for healthy individuals, 0–18 for good control [7]
MODD	Mean difference between glucose values obtained at the same time of day on two consecutive days	0.3–0.5 mmol/L for healthy individuals, 0.6–2.0 mmol/L for stable diabetes [8]
*J*-index	0.001 × (mean + SD)^2^	10 < *J* < 20 as Ideal glucose control [9]
HBGI	High blood glucose index	<4.5 for low risk of hyperglycemia [10]
LBGI	Low blood glucose index	≤1.1 for minimal risk [11]
ADRR	Range to predict both hypoglycemia and hyperglycemia	<10 for low risk [12]
GRADE score	Weighted average of glucose values, giving a greater penalties to hypoglycemia than hyperglycemia, and to more severe departures from the target level of 5.8 mmol/L	<5 reflects the euglycemic range [13]
hypoglycaemia	Percentage of GRADE score attributable to glucose values below 3.9 mmol/L	[13]
euglycaemia	Percentage of GRADE score attributable to glucose values within the range 3.9–7.8 mmol/L	[13]
hyperglycaemia	Percentage of GRADE score attributable to glucose values above 7.8 mmol/L	[13]
%CV	100 × SD/Mean	14 with range 13–16 for healthy individuals [27]
IGC	Sum of hyperglycemia index and hypoglycemia index	[14]
CONGA24	Measure of within-day glucose variability; SD of differences between any glucose value and another one exactly 24 hours latter	[14]
24 hrs-AUC	AUC attributable to glucose values above 2.2 mmol/L	135 mmol · 24 h/L for healthy individuals [20]
PT > 7.8 mmol/L	AUC attributable to glucose values above 7.8 mmol/L/24 hr-AUC	4% for healthy individuals [20, 26]
PT > 8.9 mmol/L	AUC attributable to glucose values above 8.9 mmol/L/24 hr-AUC	N/A
PT > 10 mmol/L	AUC attributable to glucose values above 10 mmol/L/24 hr-AUC	0.2 ± 0.5 for healthy individuals [20]
PT < 3.3 mmol/L	AUC attributable to glucose values within the range 2.2–3.3 mmol/L/24 hr-AUC	1 ± 1 for healthy individuals [20]

Full name of each abbreviation is given in text.

**Table 2:** CGM indicators of healthy subjects without diabetes.

	Fasting blood glucose level			
	ALL (*N* = 29)	<100 (*n* = 22)	100–126 (*n* = 7)	*P*-value
MBG (mmol/dL)	6.4 ± 0.8	6.4 ± 0.7	6.6 ± 1.1	0.471
SD (mmol/dL)	1.2 ± 0.4	1.2 ± 0.4	1.3 ± 0.4	0.562
LAGE (mmol/dL)	4.5 ± 1.6	4.4 ± 1.6	4.9 ± 1.8	0.414
MAGE (mmol/dL)	2.3 ± 0.8	2.2 ± 0.8	2.5 ± 0.7	0.388
*M*-value	7.3 ± 3.2	6.8 ± 3.1	8.8 ± 3.1	0.153
MODD (mmol/dL)	1.3 ± 0.5	1.3 ± 0.5	1.4 ± 0.5	0.593
*J*-index	19 ± 4.5	18 ± 3.9	21 ± 6.1	0.304
HBGI	0.5 (0.3, 1.0)	0.5 (0.3, 0.9)	1.0 (0.3, 1.2)	0.102
LBGI	0.4 (0.2, 1.4)	0.4 (0.2, 0.8)	0.9 (0.2, 2.0)	0.228
ADRR	4.6 (2.6, 7.3)	4.6 (2.6, 7.1)	7.7 (4.1, 11.3)	0.142
GRADE score	3.0 ± 1.3	2.7 ± 1.1	3.9 ± 1.4	0.022
hypoglycaemia	0.0 (0.0, 0.1)	0.0 (0.0, 0.1)	0.0 (0.0, 0.2)	0.446
euglycaemia	0.6 ± 0.2	0.7 ± 0.2	0.5 ± 0.2	0.044
hyperglycaemia	0.3 ± 0.2	0.3 ± 0.2	0.4 ± 0.2	0.130
CV %	19.6 (11.9, 23.8)	18.2 (11.9, 23.7)	20.7 (11.4, 24.8)	0.371
IGC	1.6 (0.7, 3.6)	1.7 (0.7, 3.6)	0.8 (0.4, 8.2)	0.460
CONGA24	1.1 ± 0.4	1.1 ± 0.4	1.1 ± 0.3	0.659
24 hrs-AUC (mmol · 24 h/dL)	155 ± 19	155 ± 19	160 ± 19	0.427
Percentage of time at glycemia (%)				
>7.8 mmol/L	16% (5%, 27%)	13% (3%, 23%)	19% (7%, 30%)	0.212
>8.9 mmol/L	1% (0%, 5%)	1% (0%, 6%)	3% (1%, 5%)	0.238
>10 mmol/L	0% (0%, 1%)	0% (0%, 1%)	0% (0%, 0%)	0.314
< 3.3 mmol/L	0% (0%, 0%)	0% (0%, 0%)	0% (0%, 2%)	0.134

Presented by mean ± SD or median (25%, 75%) depending on the normality based on Shapiro-Wilk test.

*P* value was based on either *t*-test or Wilcoxon sum rank test depending on the normality based on Shapiro-Wilk test.

**Table 3:** Correlation coefficients between GRADE score (hyopglycemia%, euglycemia%, hyperglycemia%), and HbA1c and 1,5-AG in the entire subject.

	GRADE score		Hypoglycemia%		Euglycemia%		Hyperglycemia%	
	Coefficient	*P*	Coefficient	*P*	Coefficient	*P*	Coefficient	*P*
HbA1c	0.450	0.014	−0.264	0.167	−0.191	0.321	0.396	0.034
1,5-AG	−0.583	0.001	−0.191	0.331	0.512	0.005	−0.411	0.030
